# Differences in life expectancy with and without disease using reported, measured, and combined estimates for hypertension and diabetes among older adults in Colombia

**DOI:** 10.1371/journal.pone.0349777

**Published:** 2026-06-03

**Authors:** Margarita Osuna, Liliana Ruvalcaba, Eileen Crimmins, Carlos Cano, Jennifer Ailshire

**Affiliations:** 1 Leonard Davis School of Gerontology, University of Southern California, Los Angeles,‌‌ California, United States of America; 2 Facultad de Medicina, Pontificia Universidad Javeriana, Bogotá D.C, Bogotá,‌‌ Colombia; Universidad Cooperativa De Colombia - Pasto, COLOMBIA

## Abstract

**Background:**

In Colombia, estimates of diabetes and hypertension prevalence differ substantially depending on the source of measurement. These differences have important implications for understanding disease burden in later life, particularly as the population ages and chronic conditions become more prevalent.

**Methods:**

This study uses nationally representative data from SABE-Colombia 2015 to estimate the prevalence and diseased life expectancy (DLE) with diabetes and hypertension among adults 60 and older. Prevalence was calculated using three measures: reported diagnosis, measured high-risk and a combined estimate that includes both indicators. We further disaggregated each disease into three categories: controlled, uncontrolled, and unaware. DLE was estimated using the Sullivan method.

**Results:**

For diabetes prevalence, the widest range between measures among men was at ages 80–84: 22.5% combined, 21.5% reported, and 6.7% measured. Among women the widest range occurred at ages 75–79: 34.4% combined, 32.0% reported, and 13.4% measured. An average man at age 60 was expected to live 3.8 years with diabetes using the combined measure, 3.5 years using reported diagnosis, and 1.5 years using measured data. An average women, at age 60,  had a DLE with diabetes of 5.0 years using the combined measure, 4.6 years using reported measure, and 2.0 years using measured data. For hypertension prevalence, the widest range between measures among men occurred at ages 65–69: 64.8% combined, 47.3% reported, and 32.6% measured. Among women, the widest range also occurred at ages 65–69: 74.0% combined, 63.6% reported, and 28.2% measured. At age 60, men were expected to live 13.9 years with hypertension using the combined measure, compared with 10.5 years using reported diagnosis and 8.3 years using measured data. For women, DLEat age 60 was 16.7 years combined, 14.4 years reported, and 8.0 years measured.

**Conclusion:**

We found relatively high diabetes awareness and treatment. In contrast, for hypertension, reported diagnosis underestimated both prevalence and DLE compared to combined measures.

## Introduction

Non-communicable diseases (NCDs), such as diabetes and hypertension, are among the leading causes of disability and death globally [1,2]. Current estimates indicate that hypertension affects roughly 30–50% of adults in Latin America, while about 8–13% of adults live with diabetes [3]. In Colombia, the public health burden is especially high, with NCDs accounting for nearly 76% of premature mortality [4]. At the same time, life expectancy at older ages in the country has increased in the last several decades, as much as 4.5 years for women and 7.6 years for men at age 60 [5]. The co-occurrence of rising disease prevalence and longer lives raises concerns that older adults may spend an increasing proportion of their remaining life living with chronic diseases, placing strain on individuals, families, and healthcare systems. Studies in Latin America have pointed out that a large fraction of individuals with these conditions are undiagnosed or are not adequately controlling for the disease, exacerbating the health impact of these diseases [3]. In the Colombian context, where healthcare access is uneven and health literacy is limited, it is particularly important to assess whether individuals are aware of their conditions and whether those conditions are adequately controlled. Understanding the burden of chronic disease is critical to inform effective public health planning for Colombia’s aging population.

To better understand how these conditions impact aging populations, we must consider not only their prevalence but also how long individuals live with these diseases. Disease Life Expectancy (DLE) and Disease-Free Life Expectancy (DFLE) provide complementary measures by estimating the number of years individuals are expected to live with and without disease [6]. The DLE calculation is based on disease prevalence estimates, which can vary substantially depending on the data source used to measure the disease or condition. Reported diagnoses and objective clinical measurements often yield substantially different prevalence estimates. These differences are particularly important in settings where a nontrivial proportion of the population may have undiagnosed disease. Utilizing underestimated prevalence rates to calculate DLE creates a downward bias, leading to a significant underestimation of the actual burden of disease. Existing research in Colombia has documented wide variation in reported prevalence of diabetes and hypertension, reflecting differences in study design, geographic coverage, and measurement strategies. Studies using measured blood pressure, for instance, have reported prevalence ranges for hypertension from 5–51% [7–11], while studies relying on reported diagnosis have higher estimates ranging from 47–72% [9].

### Review of prevalence of diabetes and hypertension in Colombia

While a number of studies in Colombia have estimated prevalence for diabetes and hypertension, there is wide variability in these estimates that can be largely attributed to variation in methodologies used across studies, particularly the use of measured versus reported diagnosis estimates, as well as differences in the populations studied.

Studies using survey reports have found that diabetes prevalence ranges from 1.8%−13.1% [12–14], whereas studies that used clinical measures range from 8–10.5% [13–16]. The 2010 National Demographic and Health Survey (ENDS 2010), for instance, estimated diabetes prevalence at 9.8% among adults aged 60–64 and 13.1% among those aged 70–74 [15]. Some city-level studies using reported diagnosis data report similar patterns (e.g., 12.4% in Medellín among adults aged 60–64) [14]. Studies in Bogotá that combined self-reports with HbA1c measurements found a higher prevalence when clinical data were incorporated (7.5% by self-report versus 11.0% by HbA1c) [12], demonstrating that reliance on self-reports alone can underestimate true disease burden.

Heterogeneity in estimates across studies is even more pronounced for hypertension. Administrative health records (SISPRO) – a national health information system in Colombia –reported hypertension prevalence of approximately 28% among adults aged 60 and older [10]. A population-based survey study (coordinated by the Population Health Research Institute-PURE), which covered urban and rural communities across 11 of Colombia’s most populous departments, reported a hypertension prevalence of 51.6% among adults aged 50 and older [8]. While single-city studies have reported estimates ranging from single-digit prevalence in smaller samples [7] to over 70% reported diagnosis prevalence in large urban samples [9]. For example, one single city study in Bucaramanga, a city in Colombia, reported hypertension prevalence rates of 5.0% for those aged 60–69 and 6.9% for those aged 70–79 [7]. Another study in Bogotá assessed both reported diagnosis and measured hypertension among adults aged 60 and older (N = 2,000), and found that reported diagnosis prevalence was high, ranging from 42.1% among those aged 60–64 to 72.4% among those aged 80 and older. Additionally, the study reported undiagnosed hypertension, which was considerably lower, ranging from 11.2% to 16.7% across the same age groups [9]. A systematic literature review published in 2019 highlighted substantial variation in hypertension prevalence estimates across studies in Colombia, and noted that methodological, population, and geographic differences made it hard to establish a national prevalence of the disease [17].

## Limitations to using one source of measurement

The wide variability in prevalence estimates for diabetes and hypertension highlights a key limitation: no single measurement approach fully captures the burden of these diseases among older adults in Colombia, and is therefore sensitive to access, screening, and health literacy. As a result, self-reports systematically miss individuals who have a disease or condition but have never been diagnosed [18]. In contrast, data from biomarkers or anthropometric measures provide a more objective assessment of disease status and represent the closest available approximation to a clinical gold standard in large population-based surveys. However, measured data alone cannot distinguish whether elevated risk reflects newly detected disease or poor disease management among individuals who have already been diagnosed. Combining reported diagnoses with measured indicators allows us to more accurately estimate disease prevalence. Additionally, these measures also allow us to distinguish between three meaningful disease states: (1) those who reported having a disease and measure low-risk (controlled), (2) those who report having a disease and measure high-risk (uncontrolled), and (3) those who do not report having a disease and measure high (unaware). These distinctions are rarely observed in population-based studies, where disease status is typically reduced to a single binary indicator. Yet unawareness and poor control have different implications for disease progression, healthcare utilization, and quality of life. This approach is important when estimating DLE because these estimates rely on disease prevalence and are highly sensitive to how disease is defined. Using combined measures, rather than self-reports alone, provides a more accurate estimate of the population burden and can help identify how burden of disease may vary depending on how the disease is measured.

In this study, we use nationally representative data from older adults in Colombia that include both reported doctor diagnoses and objectively measured indicators of hypertension and diabetes. While previous studies documented wide variation in prevalence estimates for these conditions, most rely on a single source of data – either reported or measured – but not both within the same population. We compare prevalence and DLE for all conditions using reported diagnosis data alone, measured high-risk of disease and a combined measure that incorporates both reported and measured information. This combined approach offers a more accurate estimate of disease burden and, in addition, allows us to better understand the proportion of individuals who are unaware of their condition, as well as those with controlled or uncontrolled disease. Our first research aim is to examine the variation of diabetes and hypertension prevalence across a reported diagnosis, measured high-risk and the combined measure. We further disaggregated the combined measure into three subgroups: those who are diagnosed and have their condition under control, whether through medications, diet and/or exercise, those who are diagnosed but have uncontrolled disease, and those who are unaware of their condition (measured high). Our second aim is to estimate DLE for both diabetes and hypertension using reported, measured, and the combined measures. DLE provides insight into the duration and potential burden of disease across the life course, offering a more nuanced picture than prevalence alone. To our knowledge, no prior studies have estimated DLE for diabetes or hypertension in Colombia. By comparing DLE estimates across sources, this study contributes a more comprehensive understanding of disease burden among older adults and fills a critical gap in the literature by integrating reported and measured data.

## Methods

### Data

We used the 2015 Colombia National Survey of Health, Well-being, and Aging “Encuesta Nacional de Salud, Bienestar y Envejecimiento” (SABE-COL), a nationally representative survey of adults ages 60 and older, to obtain disease status. Face-to-face interviews were conducted with 23,640 respondents or their proxies. A random subsample of respondents participated in a venous blood collection (N = 4,092); although fasting status was not provided in the publicly available data. Another random subsample participated in anthropometric measurements (N = 6,131). After dropping cases with missing data on disease status, the venous blood subsample consisted of 4,066 respondents, and the anthropometrics subsample consisted of 5,415 respondents. Additionally, to match the survey year, we used publicly available life tables from 2015 DANE (Departamento Administrativo Nacional de Estadistica de Colombia). DANE constructs these tables using the 2015 vital statistics, but are adjusted using the 2018 census population counts to correct for known under-registration of deaths. These census-adjusted life tables provide more accurate age-specific mortality rates than unadjusted vital statistics, ensuring a more reliable calculation of total and diseased life expectancy [19].

### Measures

*Reported disease* was based on respondents’ answers to two separate questions about prior medical diagnoses. For diabetes, respondents were asked, “Has a nurse or doctor ever told you that you have diabetes (high sugar levels in the blood)?” For hypertension, they were asked, “Has a nurse or doctor ever told you that you have high blood pressure (hypertension)?” For each condition, responses were coded as 1 if the respondent answered “yes” and 0 if they answered “no”.

*Measured disease* was determined using standard clinical thresholds for blood glucose and blood pressure. Diabetes was defined as a venous blood glucose level ≥126 mg/dL. Hypertension was defined based on the average of up to six blood pressure readings (three per arm); individuals with an average systolic blood pressure ≥140 mmHg or diastolic pressure ≥90 mmHg were classified as hypertensive.

*Combined measure of disease* includes if a respondent either reports being diagnosed with the disease and/or has measured high-risk for the disease. For diabetes, the combined measure was coded 1 if they either reported having been diagnosed with diabetes *or/and* had high measured glucose levels and coded 0 otherwise. For hypertension, the combined measure was coded 1 if the respondents either reported having been diagnosed with hypertension *or/and* had high blood pressure and coded 0 otherwise.

Using both reported diagnoses and clinical measurements, we also classified respondents into three mutually exclusive groups to describe prevalence of diseases in more detail. These subgroups were created because reported and combined prevalence estimates alone do not distinguish between individuals who are diagnosed and managing their condition versus those who remain unaware of their disease status. Disaggregating the population in this way allows for a more nuanced characterization of disease burden, capturing variation in awareness, treatment, and control that has direct implications for public health planning.

1. ***Reported and controlled***: individuals who reported a doctor’s diagnosis of either diabetes or hypertension, and did not measure high, suggesting their condition was controlled.
2. ***Reported and uncontrolled***: individuals who reported a doctor’s diagnosis of either diabetes or hypertension, but measured high, indicating uncontrolled disease.
3. ***Not reported and measured high***: individuals who did not report a doctor’s diagnosis but measured high, suggesting they are unaware or undiagnosed of their condition.

*Other variables* include sex and age categorized into 5-year age groups (60–64, 65–69, 70–74, 75–79,80–84,85+).

### Analytic strategy

First, we obtained weighted prevalences for sex-specific diabetes and hypertension by 5-year age groups using three approaches: (1) reported diagnosis alone, (2) measured high-risk disease and (3) a combined measure that classified individuals as having the condition if they either reported a diagnosis or had the disease based on measured values that exceeded clinical thresholds. Additionally, we conducted paired McNemar tests (*mcc* in Stata) to determine if differences in prevalence estimates across the three approaches were statistically significant. For all comparisons, we report both unadjusted and FDR-adjusted p-values. We did this separately for each condition (diabetes and hypertension), by 5-year age groups and sex. Then, we disaggregated disease prevalence into three subgroups: (1) reported and controlled (diagnosed but not measured high), (2) reported and uncontrolled (diagnosed and measured high), and (3) not reported and measured high (unaware). These estimates were produced separately by sex, 5-year age groups and for each condition. We conducted tests of differences of proportions to compare the three disease-status categories (controlled vs uncontrolled, controlled vs unaware, and uncontrolled vs unaware), and also report both unadjusted and FDR-adjusted p-values. All estimates were conducted using Stata 19, and all prevalence estimates were weighted to adjust for selection and non-response bias and to ensure the disease proportions were representative of the national older adult population.

To estimate DLE, we used the Sullivan method [20,21], which combines age-specific prevalence with period life table data to calculate years lived with and without disease. Person-years lived (Lx) and life expectancy (ex) for each 5-year age group and sex came from DANE’s abridged life tables [5]. We then calculated DLE for both hypertension and diabetes using SABE-Colombia prevalence estimates. For each age interval, we multiplied the prevalence of disease by the total person-years lived (Lx) to estimate the number of person-years lived with disease. From this, we determined the total years lived with disease for each age interval. DLE at age x was then calculated by dividing total years lived with disease at age X by the numbers surviving at that age (lx). Standard errors were calculated using the Sullivan variance formula, which incorporates the squared person-years lived and the variance of the age-specific prevalence estimates. Ninety-five percent confidence intervals were computed as DLE ± 1.96 × SE. Lastly, we calculated the proportion of life lived with disease by sex and for each age group by dividing the DLE by total LE at the same age. Supplemental material presents the Sullivan’s calculations. Further technical details are provided in the Health Expectancy Calculation by the Sullivan method guide [20,21]. This approach allows cross-sectional prevalence data to be integrated with demographic tools to produce health expectancy estimates that reflect the burden of disease at the population level.

## Results

**Fig 1:**
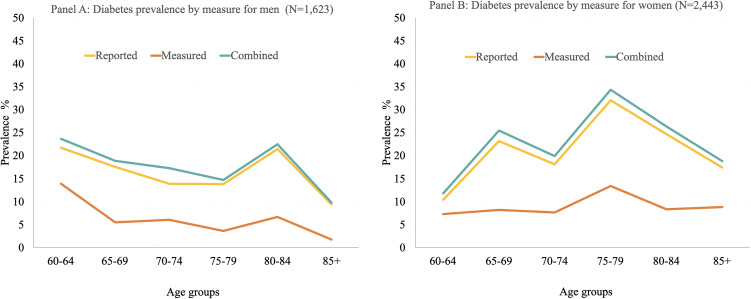
Prevalence of diabetes by measure, age-categories and sex in Colombia, SABE-COL 2015. Note: Statistically significant differences between measurement sources within each age group were assessed using McNemar tests for paired proportions. All p-values were corrected for multiple testing ‌‌using the FDR adjusted p values.

Fig 1 presents diabetes prevalence by sex and five-year age groups, using reported, measured, and combined estimates. S1 Table contains the prevalence for each measure together with the 95% Confidence intervals. Additionally, p-values from McNemar tests of differences between the measurement groups are shown in S2 Table. For both men and women, combined prevalence was significantly different from the measured prevalence across all age groups. Comparisons between reported diagnosis and measured prevalence were also statistically significant at all ages for women and at all ages for men except at ages 80–84. In contrast, differences between combined and reported diagnosis prevalence were statistically significant only at ages 60–74 for men and at ages 60–79 for women. Across all comparisons, measured prevalence was consistently lower than both reported and combined estimates. Among men (Panel A), the widest gap between measures occurred at ages 80–84, where combined prevalence was 22.5% (95% CI: 10.4–42.2), reported prevalence was 21.5% (95% CI: 9.5–41.6), and measured prevalence was 6.7% (95% CI: 2.5–16.7); which is approximately 15 percentage points difference between the combined and measured estimates. Combined prevalence was significantly different from measured at this age group. Additionally, for men, both the reported and combined measures had the highest prevalence at ages 60–64 and 80–84, with reported prevalence of 21.7% (95% CI: 12.1–36.0) at ages 60–64 and 21.5% (95% CI: 9.5–41.6) at ages 80–84, whereas the combined prevalence of 23.7% (95% CI: 13.9–37.5) at ages 60–64 and 22.5% (95% CI: 10.4–42.2) at ages 80–84.Both combined and reported prevalence were significantly different from measured prevalence. Among women (Panel B), the widest gap between measures occurred at ages 75–79. In this age group, reported prevalence was 32.0% (95% CI: 18.4–49.7), measured prevalence was 13.4% (95% CI: 5.1–30.7), and combined prevalence was 34.4% (95% CI: 20.6–51.4), with all measures being statistically different from each other.

**Fig 2:**
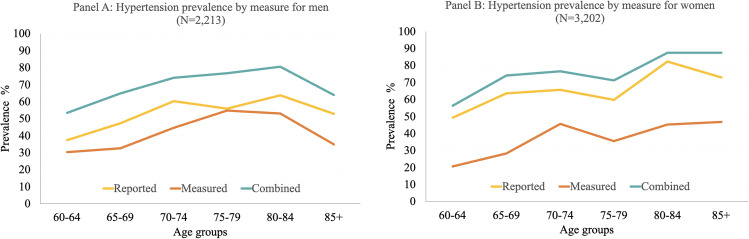
Prevalence of hypertension by measure and by age and sex in Colombia, SABE-COL 2015. Note: Statistically significant differences between measurement sources within each age group were assessed using McNemar tests for paired proportions. All p-values were corrected for multiple testing using the FDR adjusted p values.

Fig 2 presents hypertension prevalence by sex and five-year age groups using reported, measured, and combined measures. S3 Table contains the prevalence for each measure together with the 95% Confidence intervals. P-values from McNemar tests of differences between the measurement groups are shown in S4 Table. For both men and women, and across all age groups, prevalence estimates based on the combined measure were statistically significantly higher than those based on the measured estimates. Reported and measured prevalence differed significantly at ages 70–74 and older for men, for women, significant differences were found across all age groups. Differences between combined and reported diagnosis prevalence were also statistically significant across all age groups for both sexes. Among men (Panel A), the widest gap between measures occurred at ages 65–69, where combined prevalence was 64.8% (95% CI: 55.3–73.3), reported prevalence was 47.3% (95% CI: 37.8–57.1), and measured prevalence was 32.6% (95% CI: 24.2–42.2), a difference of around 32 percentage points between the combined and measured estimates. Among women (Panel B), the widest gap between measures also occurred at ages 65–69, where combined prevalence was 74.0% (95% CI: 65.6–81.0), reported prevalence was 63.6% (95% CI: 54.1–72.1), and measured prevalence was 28.2% (95% CI: 21.1–36.6), a difference of 45.8 percentage points between the combined and measured estimates.

While reported prevalence provides useful information, it does not capture whether individuals have their condition under control or remain undiagnosed. To address this, we used measured glucose and blood pressure values to further classify individuals with and without reported diagnoses of diabetes or hypertension into subgroups that reflect disease control and awareness.

**Fig 3:**
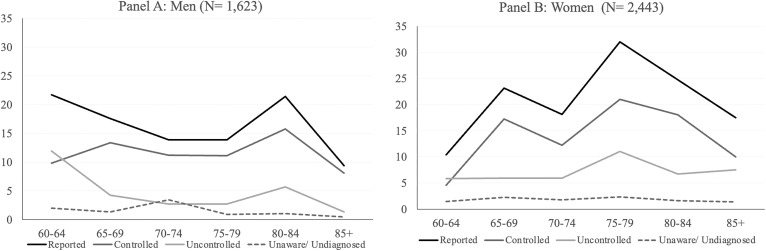
Prevalence of diabetes by control status, age and sex in Colombia, SABE-COL 2015. Note: Statistically significant differences between measurement sources within each age group were assessed using tests of differences of proportions. All p-values were corrected ‌‌for multiple testing using the FDR adjusted p values.

Fig 3 presents the prevalence of diabetes among older adults by sex and age group, disaggregated into four categories: 1) Reported, 2) Controlled, 3) Uncontrolled, and 4) Unaware. S5 Table presents the prevalence rates with 95% Confidence intervals for each age group, and for men and women separately. P-values from tests of proportions between the measurement groups are shown in S6 Table. Across most age groups and for both sexes, controlled diabetes was the most prevalent subcategory, though at ages 60–64, uncontrolled prevalence exceeded controlled for both men and women, with the lowest prevalence observed among those classified as unaware. Among men (Panel A), the widest gap between controlled and uncontrolled diabetes occurred at ages 80–84, where controlled prevalence was 15.8% (95% CI: 5.3–38.4) compared to 5.7% (95% CI: 1.8–16.3) for uncontrolled, a difference of 10.1 percentage points; however, it was not statistically significant. Controlled and uncontrolled diabetes differed significantly at all other age groups. Controlled diabetes was significantly more prevalent than unaware diabetes across all age groups, with the widest gap at ages 80–84 (15.8% controlled vs. 1.0% unaware, 14.8 percent). Differences between uncontrolled and unaware diabetes among men were statistically significant only at ages 65–69. Among women (Panel B), controlled and uncontrolled diabetes did not differ significantly at ages 60–64 (4.6% controlled vs. 5.9% uncontrolled) but were significantly different at all other age groups. The widest gap between controlled and uncontrolled prevalence among women occurred at ages 65–69, where controlled prevalence was 17.3% (95% CI: 7.0–36.5) compared to 5.9% (95% CI: 3.8–9.2) for uncontrolled. Controlled diabetes differed significantly from unaware diabetes across all age groups for women; the widest gap was at ages 75–79, where controlled prevalence was 21.0% (95% CI: 10.0–38.9) compared to 2.3% (95% CI: 0.8–6.4) unaware, a difference of 18.7 percentage points. Comparisons between uncontrolled and unaware diabetes were statistically significant at most ages, with the exception of women aged 85 and older.

**Fig 4:**
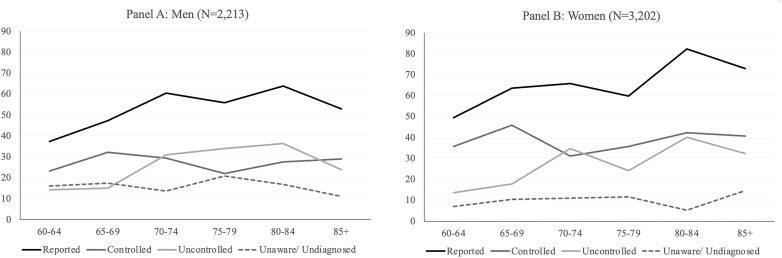
Prevalence of hypertension by control status, age and sex in Colombia, SABE-COL 2015. Note: Statistically significant differences between measurement sources within each age group were assessed using tests of differences of proportions. All p-values were corrected for multiple testing using the FDR adjusted p values.

Fig 4 presents the prevalence of hypertension among older adults by sex and age group, disaggregated into four categories: 1) Reported, 2) Controlled, 3) Uncontrolled, and 4) Unaware. S7 Table presents the prevalence rates with 95% Confidence intervals for each age group, and for men and women separately. Additionally, S8 Table presents the p-values. Among men (Panel A), differences between the controlled and uncontrolled categories were not statistically significant across most age groups (p > 0.05), with the exception of ages 75–79 (p = 0.014). In contrast, the widest gap between controlled and unaware hypertension among men occurred at ages 85 + , where controlled prevalence was 29.0% (95% CI: 15.1–48.5) compared to 11.0% (95% CI: 4.3–25.6) for unaware, a difference of 18.0 percentage points (p = 0.025). Controlled and unaware differed significantly at all age groups except 60–64. The widest gap between uncontrolled and unaware hypertension among men occurred at ages 80–84, where uncontrolled prevalence was 36.2% (95% CI: 24.9–49.3) compared to 16.8% (95% CI: 8.4–30.6) for unaware — a difference of 19.4 percentage points (p < 0.001); uncontrolled and unaware differed significantly at ages 70 and older (p < 0.05) but not at ages 60–69. Among women (Panel B), the widest gap between controlled and uncontrolled hypertension occurred at ages 65–69, where controlled prevalence was 45.8% (95% CI: 35.5–56.5) compared to 17.7% (95% CI: 12.9–24.0) for uncontrolled, which was a difference of 28.1 percentage points (p < 0.001). Controlled and uncontrolled differed significantly at ages 60–69 only (p < 0.05,), with no significant differences observed at ages 70 and older. The widest gap between controlled and unaware hypertension among women occurred at ages 80–84, where controlled prevalence was 42.2% (95% CI: 31.6–53.7) compared to 5.2% (95% CI: 2.9–9.0) for unaware (p < 0.001); both measures differed significantly across all age groups (p < 0.05). Similarly, the widest gap between uncontrolled and unaware hypertension among women occurred at ages 80–84, where uncontrolled prevalence was 40.0% (95% CI: 29.3–51.9) compared to 5.2% (95% CI: 2.9–9.0) for unaware, with a gap of almost 34.8 percentage points (p < 0.001); uncontrolled and unaware differed significantly across all age groups for women (p < 0.05).

Table 1 presents total life expectancy (LE), diseased life expectancy (DLE) and proportion of life lived with diabetes, stratified by sex, age groups, and measurement source. S9 Table contains the life table and Sullivan method results for men, and S10 Table contains the same for women. For both men and women and across all age groups, estimates based on measured disease resulted in lower DLE and a lower proportion of life lived with disease. Reported and combined measures presented higher DLE and proportion of life spent with disease. For men, at age 60, LE was 20.6 years. Using the reported disease estimate DLE was estimated at 3.5 years (proportion of life spent with disease of 16.8%) (95% CI: 3.3–3.6), whereas measured estimates DLE at 1.5 years (7.0%) (95% CI: 1.4–1.6), and the combined measure had a DLE of 3.8 years (18.5%) (95% CI: 3.7–4). At age 80, LE for men was 8.5 years. Using the reported disease estimate, DLE was estimated at 1.3 years (15.3%) (95% CI: 1.2–1.4), whereas the measured estimated DLE at 0.4 years (4.2%) (95% CI: 0.3–0.4), and the combined measure yielded 1.4 years (16.0%) (95% CI: 1.2–1.5). For women, at age 60, LE was 22.7 years. Using reported disease, DLE was estimated at 4.6 years (20.4%) (95% CI: 4.5–4.8), whereas the measured source estimated DLE at 2.0 years (8.8%) (95% CI: 1.9–2.1), and the combined measure estimated DLE to be 5.0 years (8.8%) (95% CI: 4.9–5.1). At age 80, LE for women was 8.1 years, with DLE estimated at 1.7 years (21.0%) (95% CI: 1.6–1.8) using the reported disease source, 0.7 years (8.6%) (95% CI: 0.6–0.8) using the measured source, and 1.8 years (8.6%) (95% CI: 1.7–1.8) using the combined measure.

**Table 1:** Total life expectancy (TLE), disease life expectancy (DLE) and disease-free life expectancy (DFLE) for diabetes by age for men and women, SABE-COL 2015.

			Reported Disease Status			Measured Disease Status			Combined		
	**Age**	**LE**	**DLE**	**(95% CI)**	**Proportion spent with disease (%)**	**DLE**	**(95% CI)**	**Proportion spent with disease (%)**	**DLE**	**(95% CI)**	**Proportion spent with disease (%)**
**Men** **(N = 1,623)**	60	20.6	3.5	[3.3,3.6]	16.8	1.5	[1.4,1.6]	7.0	3.8	[3.7,4]	18.5
	65	17.0	2.6	[2.5,2.7]	15.3	0.8	[0.8,0.9]	4.9	2.9	[2.7,3]	16.9
	70	13.7	2.0	[1.8,2.1]	14.4	0.6	[0.6,0.7]	4.7	2.2	[2.1,2.3]	16.1
	75	10.8	1.6	[1.5,1.7]	14.7	0.4	[0.4,0.5]	4.0	1.7	[1.5,1.8]	15.5
	80	8.5	1.3	[1.2,1.4]	15.3	0.4	[0.3,0.4]	4.2	1.4	[1.2,1.5]	16.0
	85+	6.6	0.6	[0.5,0.8]	9.4	0.1	[0.1,0.2]	1.8	0.6	[0.5,0.8]	9.8
**Women** **(N = 2,443)**	60	22.7	4.6	[4.5,4.8]	20.4	2.0	[1.9,2.1]	8.8	5.0	[4.9,2.1]	8.8
	65	18.6	4.3	[4.1,4.5]	23.2	1.7	[1.6,1.8]	9.3	4.7	[4.5,1.8]	9.3
	70	14.7	3.4	[3.3,3.6]	23.1	1.4	[1.3,1.5]	9.6	3.7	[3.5,1.5]	9.6
	75	11.2	2.9	[2.7,3]	25.5	1.2	[1.1,1.3]	10.6	3.1	[2.9,1.3]	10.6
	80	8.1	1.7	[1.6,1.8]	21.0	0.7	[0.6,0.8]	8.6	1.8	[1.7,0.8]	8.6
	85+	7.2	1.3	[1.1,1.4]	17.5	0.6	[0.5,0.8]	8.9	1.3	[1.2,0.8]	8.9

**LE:** Life Expectancy – estimates came from DANE

**DLE:** Diseeased Life Expectancy – Person-years lived with disease were calculated by multiplying disease prevalence by total person-years in each 5-year age interval. Total years lived with disease were then derived for each interval, and DLE was calculated by dividing these totals by the number surviving to the same age interval.

**Proportion spent with disease(%):** Proportion of life spent with disease – DLE divided by the total life expectancy.

Table 2 presents total life expectancy (LE), diseased life expectancy (DLE) and proportion of life lived with hypertension, stratified by sex, age groups, and measurement source. S9 Table contains the life table and Sullivan method results for men, and S10 Table contains the same for women. Across all age groups, estimates based on measured hypertension resulted in lower DLE and a lower proportion of life lived with disease, compared to estimates based on reported sources, while the combined measure produced the highest estimates. For men, at age 60, LE was 20.6 years. Using the reported disease source, DLE was estimated at 10.5 years (proportion of life spent with disease of 51.0%) (95% CI: 10.3–10.7), whereas the measured source estimated DLE at 8.3 years (40.0%) (95% CI: 8.1–8.5), and the combined measure yielded DLE of 13.9 years (67.2%) (95% CI: 13.7–14.1). At age 80, LE for men was 8.5 years. Using the reported disease source, DLE was estimated at 4.9 years (proportion of life spent with disease of 58.2%) (95% CI: 4.7–5.1), whereas the measured source estimated DLE at 3.7 years (43.7%) (95% CI: 3.5–3.9), and the combined measure yielded DLE of 6.1 years (72.1%) (95% CI: 5.9–6.3). For women, at age 60, LE was 22.7 years. Using the reported disease source, DLE was estimated at 14.4 years (63.5%) (95% CI: 14.2–14.6), whereas the measured source estimated DLE at 8.0 years (35.1%) (95% CI: 7.8–8.2), and the combined measure yielded DLE of 16.7 years (73.4%) (95% CI: 16.5–16.8). At age 80, LE for women was 8.1 years, with DLE estimated at 6.3 years (77.5%) (95% CI: 6.2–6.4) using the reported disease source, 3.7 years (46.0%) (95% CI: 3.6–3.9) using the measured source, and 7.1 years (87.5%) (95% CI: 7.0–7.2) using the combined measure. Similar to what was observed in men, measured disease source estimates consistently produced the lowest DLE values, while the combined measure resulted in the highest estimates.

**Table 2:** Total life expectancy (TLE), disease life expectancy (DLE) and disease-free life expectancy (DFLE) for hypertension, by age for men and women, SABE-COL 2015.

			Reported Disease Status			Measured Disease Status			Combined		
	**Age**	**LE**	**DLE**	**(95% CI)**	**Proportion spent with disease (%)**	**DLE**	**(95% CI)**	**Proportion spent with disease (%)**	**DLE**	**(95% CI)**	**Proportion spent with disease (%)**
**Men** **(N = 2,141)**	60	20.6	10.5	[10.3,10.7]	51.0	8.3	[8.1,8.5]	40.0	13.9	[13.7,14.1]	67.2
	65	17.0	9.4	[9.2,9.6]	55.2	7.3	[7.1,7.5]	43.0	12.1	[11.9,12.3]	71.4
	70	13.7	8.0	[7.8,8.2]	58.3	6.4	[6.2,6.6]	47.0	10.1	[9.9,10.3]	74.0
	75	10.8	6.2	[6,6.4]	57.3	5.2	[5,5.4]	48.2	8.0	[7.8,8.2]	74.0
	80	8.5	4.9	[4.7,5.1]	58.2	3.7	[3.5,3.9]	43.7	6.1	[5.9,6.3]	72.1
	85+	6.6	3.5	[3.2,3.7]	52.8	2.3	[2.1,2.5]	34.8	4.2	[4,4.4]	63.9
**Women** **(N = 3,050)**	60	22.7	14.4	[14.2,14.6]	63.5	8.0	[7.8,8.2]	35.1	16.7	[16.5,16.8]	73.4
	65	18.6	12.5	[12.3,12.7]	67.4	7.3	[7.1,7.4]	39.1	14.5	[14.3,14.6]	78.1
	70	14.7	10.1	[10,10.3]	68.8	6.3	[6.1,6.5]	43.0	11.7	[11.6,11.8]	79.5
	75	11.2	7.9	[7.7,8]	70.3	4.7	[4.5,4.9]	41.8	9.1	[8.9,9.2]	80.9
	80	8.1	6.3	[6.2,6.4]	77.5	3.7	[3.6,3.9]	46.0	7.1	[7,7.2]	87.5
	85+	7.2	5.2	[5,5.4]	73.0	3.4	[3.1,3.6]	46.8	6.3	[6.1,6.4]	87.6

**LE:** Life Expectancy – estimates came from DANE

**DLE:** Diseeased Life Expectancy – Person-years lived with disease were calculated by multiplying disease prevalence by total person-years in each 5-year age interval. Total years lived with disease were then derived for each interval, and DLE was calculated by dividing these totals by the number surviving to the same age interval.

**Proportion spent with disease(%):** Proportion of life spent with disease – DLE divided by the total life expectancy.

## Discussion

The aim of this study was to examine how different measurements of diabetes and hypertension affect prevalence and DLE estimates among older adults in Colombia. We compared estimates based on reported diagnosis, measured high-risk and a combined measure that incorporated both reported diagnosis and biomarker-based information. We found that, for both diseases, relying solely on reported diagnoses or measured estimates could underestimate prevalence and DLE compared to the combined measure. This discrepancy was especially pronounced for hypertension, a condition that is often asymptomatic and therefore more likely to go undetected without clinical screening. Further disaggregating reported diagnosis using measured biomarker data revealed additional differences in disease status. For diabetes, most individuals who reported a diagnosis had controlled disease (i.e., normal biomarker values). In contrast, for hypertension, a larger proportion of individuals who reported a diagnosis had uncontrolled blood pressure, indicating limited disease management even among those aware of their condition. Furthermore, estimates of DLE were consistently lower when calculated using reported diagnosis or measured high-risk prevalence compared to combined measures. This highlights that measurement source may fundamentally change prevalence estimates and projections of time lived with disease.

For diabetes, our prevalence estimates were slightly higher than those reported in a previous nationally representative study using ENDS 2010 [15]. Casas, (2019) reported a diabetes prevalence of 9.8% for those 60–64, whereas our estimates were higher (15.2% reported; 10.1% measured; and 16.9% combined). Among those aged 70–74, the ENDS estimate was 13%, compared to 16.5% (reported), 7.1% (measured) and 18.9% (combined) in our study. It is possible that our reported and combined estimates are higher since our data were collected five years later, and studies have highlighted that prevalence of diabetes has increased over time [22]. A recent study in Bogotá also reported a prevalence of 7.5% based on self-report and 11.0% using HbA1c measurements [12]. While this study also found a gap between measures, we cannot directly compare our results with the study, as Arteaga et al., (2024) reported results only for adults aged 18 and older combined. In contrast, our study focuses on older adults, and we observed smaller differences between reported and measured estimates.

For diabetes, the similarity between the reported disease and combined measure suggests that most older adults in Colombia are aware of their condition. It is possible that this is the case since diabetes often causes noticeable symptoms, prompting individuals to seek medical care. Although Colombia has a universal healthcare system,covering 96% of the population as of 2011 [23], barriers to access still exist. Nevertheless, data from SABE-COL suggest that diabetes management is relatively widespread: 82% of participants who reported having diabetes also reported taking medication (either insulin or oral treatments), and nearly 29% reported following a special diet. However, the presence of uncontrolled or undiagnosed cases suggests that treatment is not always adequate. For example, one study in Colombia found that the average daily dose of metformin (used to control diabetes) was lower than the internationally recommended amount, raising concerns about potential under-treatment [24]. Lastly, an important limitation of the SABE-COL survey is that participants’ fasting status was not recorded, which may have led to elevated glucose readings and potential misclassification. However, we conducted a sensitivity analysis, using a stricter glucose threshold (above 200 mg/dl), and found that even with this threshold our findings did not meaningfully change.

Our hypertension estimates are consistently higher than administrative prevalence reported in other studies, likely due to the under-registration of asymptomatic cases in clinical records. García-Peña (2022) used the SISPRO database, which only captures individuals with recorded healthcare encounters, and they reported a hypertension prevalence for adults aged 60–64 of 18.7% in 2013 and 21.1% in 2017 [10]. These results closely align with our “unaware or uncontrolled” prevalence of 25.1% for the same age group. Similarly, for those aged 80–84, García-Peña reported a 2017 prevalence of 50.8%, comparable to our unaware/uncontrolled rate of 40.1%. Differences between estimates likely reflect differences in data sources: SISPRO includes only individuals that have interacted with the healthcare system, underestimating population-level prevalence, which is particularly problematic for asymptomatic conditions like hypertension. Compared to other studies in low-and middle-income countries, our hypertension prevalence estimates among older adults are similar. For example, a nationally representative study from Brazil reported a prevalence of 66.7% of hypertension among individuals aged 60 and older [25]. Similarly, a meta-analysis of studies from African countries estimated a pooled prevalence of 55.2% among adults aged 55 and older [26]. In our study, combined prevalence among adults aged 60 and older reached 53.2% for men and 56% for women. However, at older ages, the prevalence of hypertension had a steep increase. Other studies, however, have also found that hypertension prevalence increases at older ages [27].

For hypertension, discrepancies between measures were especially pronounced among men, highlighting high underdiagnosis within this group. Although the gaps were smaller among women, differences still existed. These findings align with prior research in Colombia documenting underreporting of hypertension among older adults, even in urban areas like Bogotá with relatively broad healthcare access [9]. In part, these findings may be associated with the challenge of identifying hypertension, a condition often asymptomatic and undiagnosed until complications arise [28]. However, despite universal healthcare coverage, the Colombian health system is characterized by long wait times and accessibility barriers (i.e., restricted care delivery, regardless of coverage) [29]. Furthermore, documented difficulties in medication dispensing, including difficulty accessing medications and administrative hurdles within the insurance system, hinder consistent medication for those already diagnosed [30]. Barriers to care, together with the asymptomatic condition of hypertension, might indicate that people who have hypertension may not get appropriate treatment. This could also suggest why a significant portion of the older population may be either undiagnosed or receiving suboptimal treatment, highlighting the importance of integrating biomarkers with self-reported data to accurately estimate disease burden. Despite high rates of reported medication use in SABE-COL, many individuals—particularly those with hypertension—remained uncontrolled, reinforcing a pattern observed in high-income countries like the United States two to three decades ago, where treatment coverage did not necessarily translate into population-level control. This gap may reflect inconsistent medication adherence, suboptimal prescribing, or inadequate dosing. For example, some individuals may take medications irregularly or at lower-than-prescribed frequencies, reducing treatment effectiveness, as has been reported in studies looking at diabetes [24]. These findings point to an urgent need for interventions that go beyond access, as sustained medication use is essential for effective medical control.

Lastly, these measurement discrepancies can alter estimates of national disease burden. Our estimates show that hypertension prevalence differed substantially depending on the measurement approach used, with meaningful consequences for DLE estimates. The combined measure consistently produced the highest prevalence estimates across all age groups and for both men and women, while estimates based on reported diagnoses alone were substantially lower. These differences translated into large gaps in estimated years lived with hypertension. For example, among men aged 60, the estimated number of years lived with hypertension was 10.5 (51.0% of remaining life) based on reported data, compared to 13.9 years (67.2%) using the combined measure. A similar pattern was observed among women: at age 60, DLE was 16.7 years (64.0%) based on reported diagnosis and 19.3 years (73.9%) using the combined measure. The proportion of life spent with hypertension was also consistently higher under the combined measure, reaching nearly 88% among women aged 85 + . These results highlight the importance of integrating reported and measured data to achieve a more accurate and comprehensive estimation of hypertension prevalence. Similar patterns have been observed in Brazil, where, despite high rates of treatment, only about half of hypertensive individuals had controlled blood pressure [31]. Together, these findings highlight that access to treatment alone is not enough—accurate detection, sustained management, and integrated data sources are essential to capturing the true burden of hypertension.

Few studies have investigated DLE with diabetes and hypertension in Latin America. A study conducted by Andrade, 2009 looking at DLE with diabetes across seven major Latin American and Caribbean cities (Buenos Aires, Bridgetown, São Paulo, Santiago de Chile, Havana, and Mexico City) and Mexico found large variability across countries, using reported data. Among men aged 60, DLE ranged from 1.3 years in Havana (7.1% of remaining life with diabetes) to 4.5 years in Mexico City (22.2%), while for women it ranged from 3.1 years in Montevideo (13.5%) to 5.4 years in Bridgetown (23.7%). Our study adds two important contributions to this literature. First, we show that reported measures alone substantially underestimate the burden of disease in older Colombian adults. When clinical biomarkers are incorporated, DLE estimates increase notably, particularly for hypertension. [32]. Second, our findings highlight how measurement strategies can account for a large portion of the variability observed across studies. While Andrade’s work documents cross-country differences in DLE, it is unclear whether these reflect actual variation in disease burden or differences in diagnosis, access to care, or survey design. By directly comparing measurement strategies within one population, our results suggest that differences in how disease is captured may explain a significant part of the observed variability across settings. This highlights the need for harmonized, and different measures when comparing disease burden across Latin American countries.

This study has many strengths; we used high-quality data on disease and life expectancy that have recently become available for Colombia to provide novel estimates of DLE. Underreporting of mortality is a concern in some areas of Colombia, but we use data that have been corrected for mortality underreporting by the national statistical agency (DANE). Additionally, we use nationally representative data, allowing us to calculate prevalence and DLE estimates at the national level, rather than just in single cities, which had not been done in previous research. Another contribution of this study is that we examined how these estimates vary depending on the measurement source used, which helps provide a more comprehensive understanding of disease burden in Colombia for diabetes and hypertension. However, the present study has some limitations that should be acknowledged. First, the cross-sectional design of SABE-COL precludes the analysis of disease progression or the estimation of transition between health states. Consequently, given that the data is cross-sectional, we had to rely on the Sullivan method, which assumes that individuals with and without a specific condition face the same mortality risk. Additionally, SABE-COL surveyed only community-dwelling older adults and thus did not include the institutionalized older adult population, which tends to have the greatest care needs and may have higher disease prevalence, potentially making our estimates conservative. However, institutionalization is very rare among older Colombians [33] as few public options exist and private nursing homes are generally unaffordable. While this study provides national-level estimates for Colombia, it is important to acknowledge the country’s significant regional and socioeconomic heterogeneity. Research from the SALURBAL project has demonstrated that NCD prevalence and life expectancy vary substantially across geographical units and urban-rural contexts in Latin America [34]. Future research should utilize these data to explore how geographical disparities influence the gap between reported and measured disease burden. Such analyses will be critical for tailoring regional public health interventions but remain beyond the scope of the current national-level study. Lastly, for diabetes, it is unclear whether participants were fasting at the time of the blood draw, which may have led to overestimation of glucose levels and misclassification ‌‌of some individuals as having uncontrolled or undiagnosed diabetes.

## Conclusion

This paper examined the prevalence and DLE of diabetes and hypertension in Colombia, comparing estimates based on reported diagnoses and combined indicators. We found that prevalence and DLE estimates vary depending on the measurement source, highlighting the importance of incorporating measured data together with reported diagnosis. For diabetes, the findings showed that reported disease status provides similar results to the combined prevalence, likely due to greater awareness of the disease among older adults and widespread access to medical treatment. In contrast, for hypertension, our findings emphasize the limitations of relying solely on a single data measure, indicating that a combined measure is potentially better for estimating prevalence. The differences in prevalence and DLE estimates highlight how different measurement sources’ estimates on disease burden can vary across sources. Understanding how underreporting can occur when measuring certain diseases is important, as the burden of chronic disease continues to rise, improving measurement practices is critical to accurately tracking population health and informing equitable health policy.

## Supporting information

S1 Table Prevalence of diabetes with 95% confidence intervals and significant tests using FDR adjusted p-values for men and women based on reported, measured and combined estimates by age group.
(PDF)

S2 Table McNemar tests (unadjusted) and FDR adjusted p values comparing diabetes prevalence estimates across reported, measured, and combined definitions, by sex and age group.
(PDF)

S3 Table Prevalence of hypertension with 95% confidence intervals and significant tests using FDR adjusted p-values for men and women based on reported, measured and combined estimates by age group.
(PDF)

S4 Table McNemar tests (unadjusted) and FDR adjusted p values comparing hypertension prevalence estimates across reported, measured, and combined definitions, by sex and age group.
(PDF)

S5 Table Prevalence of diabetes with 95% confidence by control status, age and sex in Colombia, SABE-COL 2015.
(PDF)

S6 Table Pairwise tests of proportions comparing diabetes controlled, uncontrolled, and unaware, by sex and age group.
(PDF)

S7 Table Prevalence of hypertension with 95% confidence by control status, age and sex in Colombia, SABE-COL 2015.
(PDF)

S8 Table Pairwise tests of proportions comparing hypertension controlled, uncontrolled, and unaware, by sex and age group.
(PDF)

S9 Table Life table with Sullivan Method results for men for diabetes.
(PDF)

S10 Table Life table with Sullivan Method results for women for diabetes.
(PDF)

S11 Table Life table with Sullivan Method results for men for hypertension.
(PDF)

S12 Table Life table with Sullivan Method results for women for hypertension.
(PDF)
