# Supplementary material for: Differences in life expectancy with and without disease using reported, measured, and combined estimates for hypertension and diabetes among older adults in Colombia
Source: PLoS One. 2026 Jun 3;21(6):e0349777. doi: 10.1371/journal.pone.0349777 (PMC13232852; doi:10.1371/journal.pone.0349777)
Supplement: S1 Table — Prevalence of diabetes with 95% confidence intervals and significant tests using FDR adjusted p-values for men and women based on reported, measured and combined estimates by age group. (PDF) [file pone.0349777.s001.pdf]

	Men							
	Reported		Measured			Combined		
Age	%	95% CI	%		95% CI	%		95% CI
60-64	21.7	[12.1 , 36]	13.9	†	[5.8 , 29.9]	23.7	† ‡	[13.9 , 37.5]
65-69	17.6	[11.2 , 26.6]	5.6	†	[3.1 , 9.7]	18.9	† ‡	[12.3 , 28]
70-74	13.9	[9.1 , 20.6]	6.1	†	[3.3 , 11]	17.3	† ‡	[11.8 , 24.6]
75-79	13.9	[8.5 , 21.8]	3.6	†	[1.8 , 7.3]	14.8	‡	[9.2 , 22.9]
80-84	21.5	[9.5 , 41.6]	6.7		[2.5 , 16.7]	22.5	‡	[10.4 , 42.2]
85+	9.4	[4 , 20.5]	1.8	†	[0.5 , 5.9]	9.8	‡	[4.3 , 20.9]
	Women							
60-64	10.4	[7.1 , 15.1]	7.3	†	[4.6 , 11.5]	11.9	† ‡	[8.2 , 16.9]
65-69	23.2	[12.2 , 39.7]	8.2	†	[5.4 , 12.3]	25.5	† ‡	[14.3 , 41.3]
70-74	18.1	[13.1 , 24.6]	7.7	†	[5 , 11.5]	19.9	† ‡	[14.6 , 26.6]
75-79	32.0	[18.4 , 49.7]	13.4	†	[5.1 , 30.7]	34.4	† ‡	[20.6 , 51.4]
80-84	24.7	[14.4 , 39.1]	8.4	†	[4.4 , 15.4]	26.4	‡	[15.8 , 40.6]
85+	17.5	[10.2 , 28.3]	8.9	†	[3.8 , 19.2]	18.8	‡	[11.3 , 29.7]

Note: Single cross † denotes significant differences from reported based on FDR adjusted-p-value ($p<0.05$) and double cross ‡ denotes significant differences between measured and combined based on FDR adjusted-p-value ($p<0.05$)
