# Supplementary material for: Differences in life expectancy with and without disease using reported, measured, and combined estimates for hypertension and diabetes among older adults in Colombia
Source: PLoS One. 2026 Jun 3;21(6):e0349777. doi: 10.1371/journal.pone.0349777 (PMC13232852; doi:10.1371/journal.pone.0349777)
Supplement: S2 Table — McNemar tests (unadjusted) and FDR adjusted p values comparing diabetes prevalence estimates across reported, measured, and combined definitions, by sex and age group. (PDF) [file pone.0349777.s002.pdf]

Age	Reported Vs. Measured		Reported vs. Combined		Combined vs. Measured	
	Unadjusted P Value	Adjusted P Value	Unadjusted P Value	Adjusted P Value	Unadjusted P Value	Adjusted P Value
	**Men**					
60-64	0.000	0.000	0.001	0.002	0.000	0.000
65-69	0.000	0.000	0.016	0.019	0.000	0.000
70-74	0.000	0.001	0.008	0.010	0.000	0.000
75-79	0.000	0.000	0.125	0.136	0.000	0.000
80-84	0.064	0.072	0.063	0.072	0.000	0.000
85+	0.039	0.046	0.500	0.500	0.002	0.003
	**Women**					
60-64	0.000	0.000	0.000	0.000	0.000	0.000
65-69	0.000	0.000	0.000	0.000	0.000	0.000
70-74	0.000	0.000	0.004	0.005	0.000	0.000
75-79	0.000	0.000	0.004	0.005	0.000	0.000
80-84	0.000	0.000	0.250	0.257	0.000	0.000
85+	0.003	0.004	0.250	0.257	0.000	0.000
