# Supplementary material for: Differences in life expectancy with and without disease using reported, measured, and combined estimates for hypertension and diabetes among older adults in Colombia
Source: PLoS One. 2026 Jun 3;21(6):e0349777. doi: 10.1371/journal.pone.0349777 (PMC13232852; doi:10.1371/journal.pone.0349777)
Supplement: S3 Table — Prevalence of hypertension with 95% confidence intervals and significant tests using FDR adjusted p-values for men and women based on reported, measured and combined estimates by age group. (PDF) [file pone.0349777.s003.pdf]

	Men							
	Reported		Measured			Combined		
Age	%	95% CI	%		95% CI	%		95% CI
60-64	37.4	[29.8 , 45.5]	30.3		[24 , 37.5]	53.4	† ‡	[45.2 , 61.5]
65-69	47.3	[37.8 , 57.1]	32.6		[24.2 , 42.2]	64.8	† ‡	[55.3 , 73.3]
70-74	60.4	[49.6 , 70.3]	44.6	†	[33.6 , 56.2]	74.0	† ‡	[64 , 82]
75-79	55.9	[42.9 , 68.1]	54.8	†	[42 , 67]	76.7	† ‡	[66.5 , 84.6]
80-84	63.8	[50.8 , 75.1]	53.0	†	[40 , 65.6]	80.6	† ‡	[70.7 , 87.8]
85+	52.8	[33.2 , 71.6]	34.8	†	[20.5 , 52.5]	63.9	† ‡	[41 , 81.8]
	**Women**							
60-64	49.4	[42.1 , 56.7]	20.6	†	[16.4 , 25.6]	56.4	† ‡	[48.6 , 63.9]
65-69	63.6	[54.1 , 72.1]	28.2	†	[21.1 , 36.6]	74.0	† ‡	[65.6 , 81]
70-74	65.6	[57.8 , 72.7]	45.6	†	[37.3 , 54.2]	76.6	† ‡	[69.7 , 82.4]
75-79	59.8	[47.3 , 71.1]	35.5	†	[26.3 , 46]	71.3	† ‡	[57.5 , 82]
80-84	82.3	[74.5 , 88.1]	45.2	†	[34.2 , 56.6]	87.4	† ‡	[80 , 92.4]
85+	73.0	[61 , 82.3]	46.8	†	[33.8 , 60.4]	87.6	† ‡	[78.6 , 93.1]

Note: Single cross † denotes significant differences from reported based on FDR adjusted-p-value ($p<0.05$) and double cross ‡ denotes significant differences between measured and combined based on FDR adjusted-p-value ($p<0.05$)
