# Supplementary material for: Differences in life expectancy with and without disease using reported, measured, and combined estimates for hypertension and diabetes among older adults in Colombia
Source: PLoS One. 2026 Jun 3;21(6):e0349777. doi: 10.1371/journal.pone.0349777 (PMC13232852; doi:10.1371/journal.pone.0349777)
Supplement: S5 Table — Prevalence of diabetes with 95% confidence by control status, age and sex in Colombia, SABE-COL 2015. (PDF) [file pone.0349777.s005.pdf]

	Men								Women							
	Reported		Controlled		Uncontrolled		Unaware/ Undiagnosed		Reported		Controlled		Uncontrolled		Unaware/ Undiagnosed	
Age	%	95% CI	%	95% CI	%	95% CI	%	95% CI	%	95% CI	%	95% CI	%	95% CI	%	95% CI
60-64	21.7	[12.1 , 36.0]	9.8	[5.4 , 17.2]	11.9	[4.3 , 29.2]	2.0	[0.9 , 4.1]	10.4	[7.1 , 15.1]	4.6	[2.8 , 7.3]	5.9	[3.5 , 9.7]	1.4	[0.5 , 3.7]
65-69	17.6	[11.2 , 26.6]	13.4	[7.7 , 22.3]	4.2	[2.2 , 8.1]	1.3	[0.5 , 3.5]	23.2	[12.2 , 39.7]	17.3	[7.0 , 36.5]	5.9	[3.8 , 9.2]	2.3	[1.0 , 5.0]
70-74	13.9	[9.1 , 20.6]	11.2	[6.9 , 17.7]	2.7	[1.2 , 5.8]	3.4	[1.4 , 8.1]	18.1	[13.1 , 24.6]	12.3	[8.0 , 18.4]	5.9	[3.7 , 9.2]	1.8	[0.7 , 4.5]
75-79	13.9	[8.5 , 21.8]	11.1	[6.5 , 18.4]	2.7	[1.2 , 6.1]	0.9	[0.2 , 3.4]	32.0	[18.4 , 49.7]	21.0	[10.0 , 38.9]	11.1	[3.5 , 30.0]	2.3	[0.8 , 6.4]
80-84	21.5	[9.5 , 41.6]	15.8	[5.3 , 38.4]	5.7	[1.8 , 16.3]	1.0	[0.3 , 3.3]	24.7	[14.4 , 39.1]	18.0	[8.7 , 33.6]	6.7	[3.2 , 13.4]	1.6	[0.4 , 6.3]
85+	9.4	[4.0 , 20.5]	8.1	[3.1 , 19.2]	1.3	[0.3 , 5.9]	0.4	[0.1 , 2.2]	17.5	[10.2 , 28.3]	10.0	[5.2 , 18.2]	7.5	[2.9 , 18.2]	1.4	[0.4 , 5.0]
