# Supplementary material for: Differences in life expectancy with and without disease using reported, measured, and combined estimates for hypertension and diabetes among older adults in Colombia
Source: PLoS One. 2026 Jun 3;21(6):e0349777. doi: 10.1371/journal.pone.0349777 (PMC13232852; doi:10.1371/journal.pone.0349777)
Supplement: S6 Table — Pairwise tests of proportions comparing diabetes controlled, uncontrolled, and unaware, by sex and age group. (PDF) [file pone.0349777.s006.pdf]

	Controlled vs Uncontrolled		Controlled vs unaware		Uncontrolled vs. Unaware	
	Unadjusted P Value	Adjusted P Value	Unadjusted P Value	Adjusted P Value	Unadjusted P Value	Adjusted P Value
**Age**			**Men**			
60-64	0.030	0.044	0.000	0.000	0.072	0.089
65-69	0.031	0.044	0.000	0.000	0.004	0.012
70-74	0.020	0.031	0.000	0.001	0.137	0.159
75-79	0.007	0.017	0.000	0.000	0.066	0.085
80-84	0.114	0.137	0.033	0.044	0.556	0.589
85+	0.018	0.029	0.018	0.029	1.000	1.000
			**Women**			
60-64	0.625	0.643	0.000	0.000	0.000	0.000
65-69	0.495	0.000	0.000	0.000	0.000	0.000
70-74	0.032	0.044	0.000	0.000	0.000	0.000
75-79	0.012	0.023	0.000	0.000	0.017	0.029
80-84	0.014	0.026	0.000	0.000	0.011	0.023
85+	0.008	0.018	0.001	0.003	0.473	0.533
