# Supplementary material for: Differences in life expectancy with and without disease using reported, measured, and combined estimates for hypertension and diabetes among older adults in Colombia
Source: PLoS One. 2026 Jun 3;21(6):e0349777. doi: 10.1371/journal.pone.0349777 (PMC13232852; doi:10.1371/journal.pone.0349777)
Supplement: S7 Table — Prevalence of hypertension with 95% confidence by control status, age and sex in Colombia, SABE-COL 2015. (PDF) [file pone.0349777.s007.pdf]

	**Men**								**Women**							
	Reported		Controlled		Uncontrolled		Unaware/ Undiagnosed		Reported		Controlled		Uncontrolled		Unaware/ Undiagnosed	
Age	%	95% CI	%	95% CI	%	95% CI	%	95% CI	%	95% CI	%	95% CI	%	95% CI	%	95% CI
60-64	37.4	[29.8 , 45.5]	23.1	[16.6 , 31.2]	14.2	[9.8 , 20.1]	16.1	[11.8 , 21.6]	49.4	[42.1 , 56.7]	35.8	[29.4 , 42.7]	13.6	[10.4 , 17.6]	7.0	[4.7 , 10.3]
65-69	47.3	[37.8 , 57.1]	32.2	[23.4 , 42.5]	15.1	[10.6 , 21.2]	17.5	[10.5 , 27.6]	63.6	[54.1 , 72.1]	45.8	[35.5 , 56.5]	17.7	[12.9 , 24.0]	10.5	[6.0 , 17.6]
70-74	60.4	[49.6 , 70.3]	29.4	[21.1 , 39.4]	31.0	[20.4 , 44.0]	13.6	[8.1 , 21.9]	65.6	[57.8 , 72.7]	31.1	[25.1 , 37.7]	34.6	[26.2 , 44.0]	11.0	[6.8 , 17.3]
75-79	55.9	[42.9 , 68.1]	22.0	[15.0 , 31.0]	33.9	[20.3 , 50.9]	20.9	[12.7 , 32.2]	59.8	[47.3 , 71.1]	35.7	[26.5 , 46.2]	24.0	[17.4 , 32.2]	11.5	[5.7 , 21.9]
80-84	63.8	[50.8 , 75.1]	27.6	[16.9 , 41.6]	36.2	[24.9 , 49.3]	16.8	[8.4 , 30.6]	82.3	[74.5 , 88.1]	42.2	[31.6 , 53.7]	40.0	[29.3 , 51.9]	5.2	[2.9 , 9.0]
85+	52.8	[33.2 , 71.6]	29.0	[15.1 , 48.5]	23.8	[13.1 , 39.3]	11.0	[4.3 , 25.6]	73.0	[61.0 , 82.3]	40.7	[27.4 , 55.6]	32.2	[21.4 , 45.3]	14.6	[8.0 , 25.2]
