# Supplementary material for: Differences in life expectancy with and without disease using reported, measured, and combined estimates for hypertension and diabetes among older adults in Colombia
Source: PLoS One. 2026 Jun 3;21(6):e0349777. doi: 10.1371/journal.pone.0349777 (PMC13232852; doi:10.1371/journal.pone.0349777)
Supplement: S8 Table — Pairwise tests of proportions comparing hypertension controlled, uncontrolled, and unaware, by sex and age group. (PDF) [file pone.0349777.s008.pdf]

	**Controlled vs Uncontrolled**		**Controlled vs unaware**		**Uncontrolled vs. Unaware**	
	Unadjusted P Value	Adjusted P Value	Unadjusted P Value	Adjusted P Value	Unadjusted P Value	Adjusted P Value
**Age**			**Men**			
60-64	0.310	0.348	1.000	1.000	0.310	0.348
65-69	0.245	0.316	0.002	0.004	0.053	0.079
70-74	0.543	0.543	0.000	0.000	0.000	0.000
75-79	0.856	0.014	0.009	0.014	0.005	0.009
80-84	0.246	0.316	0.007	0.013	0.000	0.000
85+	0.395	0.431	0.016	0.025	0.001	0.002
			**Women**			
60-64	0.000	0.000	0.000	0.000	0.000	0.000
65-69	0.000	0.000	0.000	0.000	0.000	0.000
70-74	0.070	0.100	0.000	0.000	0.000	0.000
75-79	0.147	0.203	0.000	0.000	0.000	0.000
80-84	0.280	0.348	0.000	0.000	0.000	0.000
85+	0.295	0.348	0.000	0.000	0.000	0.000
