# Supplementary material for: Differences in life expectancy with and without disease using reported, measured, and combined estimates for hypertension and diabetes among older adults in Colombia
Source: PLoS One. 2026 Jun 3;21(6):e0349777. doi: 10.1371/journal.pone.0349777 (PMC13232852; doi:10.1371/journal.pone.0349777)
Supplement: S10 Table — Life table with Sullivan Method results for women for diabetes. (PDF) [file pone.0349777.s010.pdf]

				Self Reported				Measured				Combined			
Age group	Numbers surviving to age x	Person years lived in age interval	Total life expectancy	Diseased Life Expectancy	Proportion of life with disease	DLE Lower Confidence Interval	DLE Upper Confidence Interval	Diseased Life Expectancy	Proportion of life with disease	DLE Lower Confidence Interval	DLE Upper Confidence Interval	Diseased Life Expectancy	Proportion of life with disease	DLE Lower Confidence Interval	DLE Upper Confidence Interval
x - x+n	lx	nLx	ex	DLE	%dle/tle			DLE	%dle/tle			DLE	%dle/tle		
60-64	100000	490481	22.70	4.63	20.41	4.47	4.79	2.01	8.84	1.89	2.12	5.05	22.24	4.89	5.21
65-69	95889.67	464377	18.55	4.30	23.16	4.14	4.45	1.72	9.26	1.61	1.83	4.66	25.10	4.50	4.82
70-74	89395.05	423645	14.71	3.40	23.14	3.25	3.55	1.42	9.63	1.31	1.52	3.67	24.97	3.52	3.82
75-79	79415.03	362913	11.22	2.86	25.52	2.72	3.01	1.19	10.57	1.08	1.29	3.07	27.36	2.92	3.22
80-84	65010.55	258397	8.12	1.71	21.03	1.57	1.84	0.70	8.61	0.60	0.79	1.83	22.53	1.69	1.97
85+	37652.99	269718	7.16	1.25	17.48	1.08	1.42	0.63	8.86	0.51	0.76	1.35	18.84	1.17	1.53

Note: Data for the central death rate (nMx) come directly from the DANE (Departamento Administrativo Nacional de Estadistica) life tables from the year 2015. DLE and the variance/standard error terms, were calculated following the Sullivan method.
