# Supplementary material for: Differences in life expectancy with and without disease using reported, measured, and combined estimates for hypertension and diabetes among older adults in Colombia
Source: PLoS One. 2026 Jun 3;21(6):e0349777. doi: 10.1371/journal.pone.0349777 (PMC13232852; doi:10.1371/journal.pone.0349777)
Supplement: S11 Table — Life table with Sullivan Method results for men for hypertension. (PDF) [file pone.0349777.s011.pdf]

				Self Reported				Measured				Combined			
Age group	Numbers surviving to age x	Person years lived in age interval	Total life expectancy	Diseased Life Expectancy	Proportion of life with disease	DLE Lower Confidence Interval	DLE Upper Confidence Interval	Diseased Life Expectancy	Proportion of life with disease	DLE Lower Confidence Interval	DLE Upper Confidence Interval	Diseased Life Expectancy	Proportion of life with disease	DLE Lower Confidence Interval	DLE Upper Confidence Interval
x - x+n	lx	nLx	ex	DLE	%dle/tle			DLE	%dle/tle			DLE	%dle/tle		
60-64	100000	483975	20.64	10.54	51.05	10.34	10.74	8.26	52.02	10.74	10.74	13.87	103.89	10.74	10.74
65-69	93157.538	442358	16.96	9.37	55.25	9.18	9.56	7.29	56.39	9.56	9.56	12.11	104.23	9.56	9.56
70-74	83227.1579	383694	13.67	7.97	58.32	7.78	8.16	6.43	59.70	8.16	8.16	10.11	104.87	8.16	8.16
75-79	69658.0617	307211	10.82	6.20	57.26	6.01	6.39	5.22	59.02	6.39	6.39	8.01	106.37	6.39	6.39
80-84	52801.4294	218403	8.46	4.92	58.20	4.72	5.13	3.70	60.62	5.13	5.13	6.10	108.68	5.13	5.13
85+	34597.9915	228308	6.60	3.49	52.81	3.24	3.73	2.30	56.46	3.73	3.73	4.21	114.84	3.73	3.73

Note: Data for the central death rate (nMx) come directly from the DANE (Departamento Administrativo Nacional de Estadistica) life tables from the year 2015.
DLE and the variance/standard error terms, were calculated following the Sullivan method.
