# Supplementary material for: Differences in life expectancy with and without disease using reported, measured, and combined estimates for hypertension and diabetes among older adults in Colombia
Source: PLoS One. 2026 Jun 3;21(6):e0349777. doi: 10.1371/journal.pone.0349777 (PMC13232852; doi:10.1371/journal.pone.0349777)
Supplement: S12 Table — Life table with Sullivan Method results for women for hypertension. (PDF) [file pone.0349777.s012.pdf]

| | | | | Self Reported | | | | Measured | | | | Combined | | | |
|---|---|---|---|---|---|---|---|---|---|---|---|---|---|---|---|
| Age group | Numbers surviving to age x | Person years lived in age interval | Total life expectancy | Diseased Life Expectancy | Proportion of life with disease | DLE Lower Confidence Interval | DLE Upper Confidence Interval | Diseased Life Expectancy | Proportion of life with disease | DLE Lower Confidence Interval | DLE Upper Confidence Interval | Diseased Life Expectancy | Proportion of life with disease | DLE Lower Confidence Interval | DLE Upper Confidence Interval |
| x - x+n | lx | nLx | ex | DLE | %dle/tle | | | DLE | %dle/tle | | | DLE | %dle/tle | | |
| 60-64 | 100000 | 490481 | 22.70 | 14.42 | 63.53 | 14.24 | 14.60 | 7.97 | 35.13 | 7.79 | 8.16 | 16.66 | 73.41 | 16.50 | 16.8234435 |
| 65-69 | 95889.6659 | 464377 | 18.55 | 12.51 | 67.44 | 12.34 | 12.68 | 7.26 | 39.13 | 7.08 | 7.44 | 14.49 | 78.09 | 14.34 | 14.6395683 |
| 70-74 | 89395.0494 | 423645 | 14.71 | 10.12 | 68.80 | 9.95 | 10.28 | 6.32 | 42.99 | 6.14 | 6.50 | 11.70 | 79.53 | 11.55 | 11.8361033 |
| 75-79 | 79415.0294 | 362913 | 11.22 | 7.89 | 70.29 | 7.73 | 8.04 | 4.69 | 41.76 | 4.52 | 4.85 | 9.08 | 80.90 | 8.95 | 9.20690808 |
| 80-84 | 65010.545 | 258397 | 8.12 | 6.30 | 77.52 | 6.16 | 6.44 | 3.74 | 46.04 | 3.57 | 3.91 | 7.11 | 87.52 | 7.00 | 7.22146796 |
| 85+ | 37652.9948 | 269718 | 7.16 | 5.23 | 72.96 | 5.03 | 5.43 | 3.36 | 46.84 | 3.13 | 3.58 | 6.27 | 87.58 | 6.13 | 6.42181579 |

**Note:** Data for the central death rate (nMx) come directly from the DANE (Departamento Administrativo Nacional de Estadistica) life tables from the year 2015.
DLE and the variance/standard error terms, were calculated following the Sullivan method.
